# Increased breastfeeding; an educational exchange program between India and Norway improving newborn health in a low- and middle-income hospital population

**DOI:** 10.1186/s41043-022-00297-8

**Published:** 2022-05-03

**Authors:** Kirsti Haaland, Sadasivam Sitaraman

**Affiliations:** 1 Department of Global Health, Oslo University Hospital, Ullevål, Kirkeveien 166, 0450 Oslo, Norway; 2 Department of Neonatal Intensive Care, Division of Paediatric and Adolescent Medicine, Oslo University Hospital, Ullevål, Kirkeveien 166, 0450 Oslo, Norway; 3 Sir Padampat. I.N.C.H. S.M.S. Medical College, Jaipur, India

**Keywords:** Human milk, Milk bank, Hygiene, Nurse empowerment, Collaboration, Health literacy

## Abstract

**Background:**

The purpose of the project was to improve newborn health in neonatal care units in a low resource area with high neonatal mortality, predominantly by better nutrition and educational exchange of health care workers.

**Method:**

A fourfold program to make human milk production and distribution feasible and desirable. 1 Education to enlighten health care workers and parents to the excellence of human milk. 2 Lactation counselling to address the various challenges of breastfeeding. 3 Improving infants´ general condition. 4 Infrastructure alterations in the hospital. A collaboration between hospitals in India and Norway.

**Results:**

The number of infants receiving human milk increased pronouncedly. Systematic, professional lactation counselling, the establishment of a milk bank, and empowerment of nurses was perceived as the most important factors.

**Conclusions:**

It is possible to greatly improve nutrition and the quality of newborn care in low/middle income settings by optimising human resources. Viable improvements can be obtained by long-term health partnership, by involving all hierarchal levels and applying locally developed customized methods.

**Supplementary Information:**

The online version contains supplementary material available at 10.1186/s41043-022-00297-8.

## Background

In 2010, the world’s neonatal mortality rate (NMR) had declined slowly (28% the last 20 years) to 23 deaths in the first 28 days of living per 1000 live births. The progress was slowest in the regions with the highest rates [1]. At that time, the NMR in Rajasthan, the largest state in India was 40, in Norway 1.9. As the prime ministers of India and Norway in 2006 had made commitments to collaborate towards achieving Millennium Development Goal 4: Reduce child mortality, a partnership agreement was signed between neonatal wards in a Rajasthan and a Norwegian hospital to increase the quality of newborn care [2].

To secure surviving, optimally developed, healthy children, neonatal nutrition is almost as essential as respiration and circulation [3]. Human milk contains all essential nutrients and the composition in the mothers´ milk is customized by nature according to the gestational age of the infant [4]. Furthermore, human milk provides antimicrobial protection not present in formula and it is better tolerated [5, 6]. Human milk is by far the best nourishment for the newborn [7].

Why do not all newborn babies receive their mothers’ milk? Two main reasons are lack of knowledge about the superiority of human milk over all alternatives and practical challenges of suckling and enteral feeding [8–11]. For hospitalised infants, there may also be infrastructural limitations.

This project aimed to increase health literacy, practical skills and infrastructure, in order to make milk production and distribution desirable and feasible.

## Methods

The project was a collaboration between S.M.S. Medical College with the attached hospitals J K Lone and Mahila Chikitsalaya in Rajasthan, India, and Oslo University Hospital, Norway. J K Lone is a paediatric hospital (level II, changing to level III during the project), all neonates being referred from extramural units, covering more than 40,000 annual deliveries. Bed strength was originally 45 in the neonatal units Special Newborn Care Unit (SNCU) (for those not in need of respiratory support) and Neonatal Intensive Care Unit (NICU). Mahila Chikitsalaya is mainly an obstetrical hospital with 17,000 deliveries/year, and neonatal bed strength of 43 (no invasive respiratory care). Oslo University Hospital is level IIIb with 47 neonatal beds.

The main project period was 2013–2016, with scaled-down maintenance the following three years.

### Human resources

Before the initiation of the project, parents were not admitted into the neonatal wards. Four nurses were exchanged every 6 month between the neonatal units in India and Norway. After the first 3 years, the presence of Norwegian nurses was infrequent, whereas the group of Indian nurses trained in Norway kept expanding by 2 every half year. Three Indian paediatricians visited the Norwegian unit for 6 months each as observers. A Norwegian neonatologist held the academic responsibility. She supervised the Indians in Norway and visited the Indian unit for 14 days twice a year, in addition to web-based contact all 6 years.

The head of the milk bank in the Norwegian hospital spent approximately ten months in the Indian hospitals over the first 5 years. A devoted Indian senior consultant was appointed to head a new lactation centre with a milk bank. Full-time lactation counsellors and a technical assistant were also employed.

Department of Global Health, Oslo University Hospital conducted the overall administration of the project and visited India at least once a year. To facilitate the work of the nurses and secure momentum of the project, the office and the academic responsible physician had a continuous dialogue with the heads of the departments and the hospital administrations and met with the health authorities in the state of Rajasthan.

### Interventions

A fourfold program was developed (Table 1).

**Table 1:** The fourfold program to make human milk production and distribution feasible and desirable

1	Education to enlighten health care workers and parents to the excellence of human milk
2	Lactation counselling to address the various challenges of breastfeeding
3	Improving infants´ general condition
4	Infrastructure alterations in the hospital (I-V)

#### Education to enlighten health care workers and parents to the excellence of human milk

In Norway, the Indian nurses worked in a large neonatal unit with a strong focus on nutrition. They were exposed to a culture where the attitude to human milk and possible ways of feeding, makes human milk the obvious first choice. They experienced short-term advatages and learned about the long-term advantages of this practice. They obtained practical experience in assisting mothers with breastfeeding and expression of milk by hand or mechanical pumps and in feeding by cup and tube.

The Norwegian nurses in India had extended knowledge of intensive newborn care and all aspects of lactation. They provided theoretical and practical education individually, in small groups, seminars and workshops.

All nurses and doctors were trained and empowered to share knowledge.

The first lactation counsellors were, educated by head of the milk bank Oslo, Norwegian nurses and head of the new lactation centre. They were then entrusted with the main responsibility of providing mothers with knowledge that made them *want* to breastfeed. Every day all maternity and neonatal wards were visited by counsellors. In the later phase of the project all counsellors attended standardised eight hour theory and 32 h hands-on training courses.

To further improve and encourage the activity of all staff, follow-up regular training sessions were organized internally and with visiting specialists, giving lectures on the topic.

#### Lactation counselling to address the various challenges of breastfeeding

After convincing the mothers about the benefits of human milk, the counsellors provided general advice and mental support to enable lactation. Mothers who experienced problems were invited for individual guidance the same day.

#### Improving infants’ general condition

Indians and Norwegians worked closely together bedside, thereby quickly discovered and addressed procedures and systems possible to alter in order to improve care. Better nursing care was obtained mainly by empowering nurses in their skills and in their belief in the effect of their work. Teaching relevant theory and supervising bedside hands-on training in pain relief, developmental care and infection control were performed to minimise neonates’ energy expenditure, avoid infections and enhance their ability to tolerate enteral feeds and to suckle.

For better observations of the patients and relation to their families, every shift each infant was assigned a responsible nurse. “Nurses’ daily record” documenting nutrition, weight gain, temperature etc., was developed.

#### Infrastructure alterations in the hospital

All suggested alterations were discussed with nurses, doctors and head of the department, major changes also with the hospital administration.

**I-Bringing mothers and their sick or preterm neonates together.**

To allow mothers into the neonatal units, advantages, requirements, safety and precautions were thoroughly discussed with hospital authorities and all staff. Doctors persuaded the mother and the decision-maker in the family of the importance of mother and infant being together. Nurses facilitated physical contact between mother and infant. Depending on the condition of the infant the mother would touch, help change the diaper, sponge, feed or give kangaroo mother care (KMC). Mothers were provided with a place to sleep, eat and wash. All staff encouraged each other to respectfully welcome all mothers.

**II-Defining human milk as the default nourishment.**

In order to initiate prolactin secretion and thus early lactation, routines were established in collaboration with the maternity ward to help newborns to the breast within one hour of delivery. Doctors were committed to explicitly prescribe human milk whenever possible and nurses to strive to feed the right amount at the right time by optimal means and to document this. Mothers were urged to always provide fresh milk and contact the counsellors if needed.

**III-Establishment of a lactation centre with milk bank and mil kitchens.**

Some mothers produce milk in abundance, others are not able to produce the adequate amount for their baby, especially not the first days. To provide human milk for all neonates in the hospitals, a lactation centre with counsellors and milk bank as well as milk kitchens were established.

The centre accomodated room for individual, undisturbed counselling. The mother-infant dyads were observed in the feeding situation and tutored according to their needs. Mothers whose infants were not able to nurse from the breast were taught how to express milk by hand and by a mechanical pump.

In order to provide for the newborns who could not receive enough milk from their mothers, a milk bank was established. When a mother produced more milk than required by her infant, she was informed about the possibility to donate her surplus. This was the first milk bank in Rajasthan and several actions enhanced a positive attitude towards it. The inauguration was celebrated with the presence of the state health minister and broadcast in several media as an important advancement for Indian healthcare. A father of a healthy infant went around the hospital promoting the milk bank to the other fathers who then encouraged their wives. A member of staff with a healthy infant donated her milk.

Locally customized, detailed protocols were compiled describing the routines for handling milk and equipment (later contributing to national guidelines [12]). There was intense initial and regular training including technical procedures such as collection, pasteurization and storage as well as hygiene, quality control and tracing. Milk was daily transported in sealed bottles in a cool bag from the bank to the different units´ milk kitchens. To avoid waste and shortage, a system was established to keep track of the amount of milk each infant required and consumed every day.

**IV-Infection control.**

In order to improve hand hygiene, every person entering the units receive individual instructions to remove jewellery, sacred threads, watches and similar and perform hand wash. Washbasins and soap were to be made accessible for everyone, and all reusable towels removed. Rubbing alcohol was to be available at every bedside.

Instead of sending linen to the hospital laundry where it would be dried on the ground outside among cars and livestock, it was organized for washing and drying inside the NICU.

Protocols for regular cleaning of all equipment were developed.

To limit the spread of infections from the older patients, doctors from the seven paediatric wards attending “their” neonates were rendered superfluous by one senior doctor taking the main responsibility for all patients in the NICU.

**V-Assembling and increasing the number of neonatal beds.**

Because of an inadequate number of beds in the neonatal units, all paediatric wards had a “neonatal cubicle”. Discussion with authorities was held with strong advocacy for the allocation of more beds and dedicated human resources to this group of patients.

Rounds were reorganized to facilitate information between doctors, nurses and parents.

### Data collection

All exchanged health workers answered a standard questionnaire (Additional file 1: Supplement 1) mid- and end period providing qualitative description and evaluation of the project including changes and achievement. Data on milk consumption and hand hygiene were retrieved from repeated structured three-day open observations in the units performed by the Norwegian nurses (Additional file 2: Supplement 2). Quantitative data on counselling and donated milk were obtained from lactation centre protocols. Nature of feed at discharge from two neonatal units after Caesarean section was recorded three months in 2015. Both units received milk from the bank, but as only one had project nurses and -counsellors, the impact of this could be suggested.

The project was not designed for research. There is a lack of structured data collection and large changes over time in the population. As advanced statistics is thus unfeasible, numbers are offered merely to create a picture of the situation.

The work was conducted following prevailing ethical principles.

## Results

### The number of infants receiving human milk increased (Fig. 1)

**Fig. 1:**
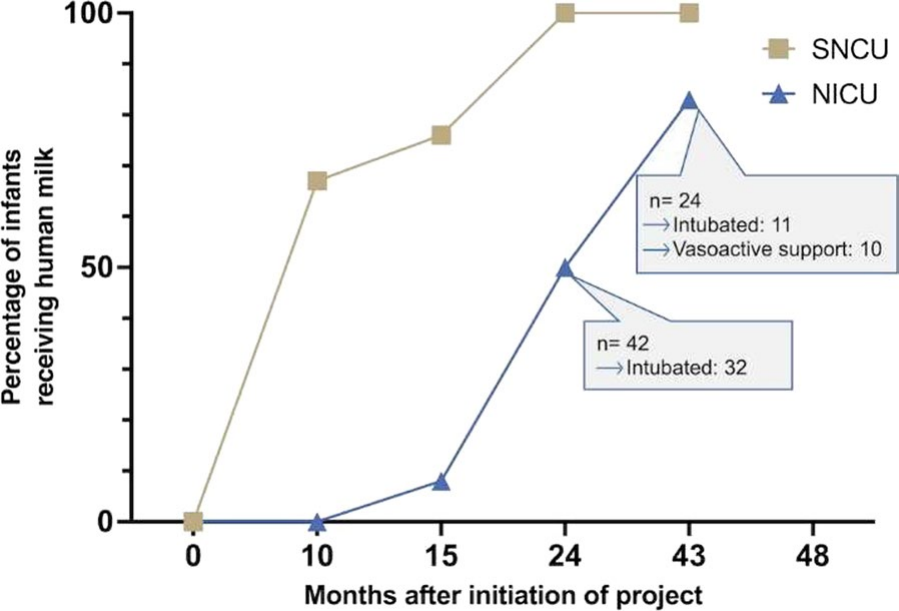
Data from three-days surveys. Two years into the project, when the lactation centre was well established, *all* infants in the SNCU and 50% in the NICU received human milk and none received formula. Seven months after the end of the 3-year continuous exchange of nurses, only four infants did not receive any enteral feed

In the initial phase of the project, a few of the healthiest babies were infrequently breastfed behind a curtain in the hall outside the unit. Some infants were fed by cup or tube, mainly diary milk or formula. Most patients in the SNCU and all in the NICU were on “nothing by mouth". Their nutrition consisted of intravenously administered glucose. Increase in the number of infants receiving human milk were first seen in the SNCU were the measures to secure the infants the presence of their mother were first implemented. In this unit, almost 50% also nursed at least partly from the breast 15 months into the project.

### Professional counselling made a difference

**Fig. 2:**
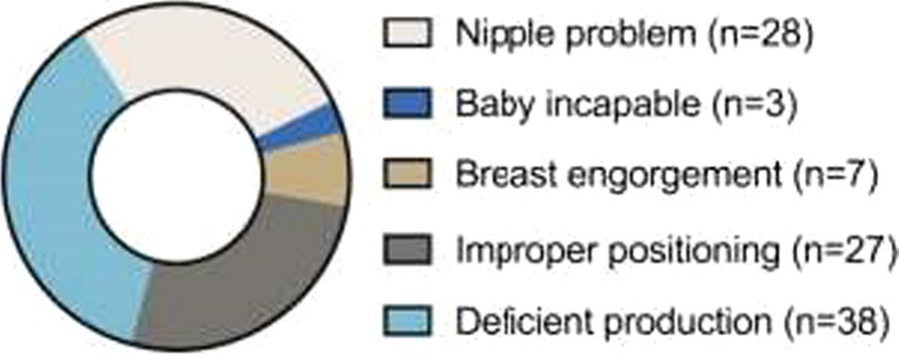
The most common lactation problems in 155 patients in a 12-bed low dependency neonatal care unit at Mahila hospital (January–June 2016)

The lactation centre Jeevan Dhara was inaugurated in March 2015. In less than two years, 15,000 mothers were counselled. Most problems preventing lactation were easily overcome. In the neonatal units, the most common challenge was the infants being too sick or premature to feed on the breast. Teaching mothers extraction of milk, by hand or pump, enabled production and prevented breast engorgement. In the maternity wards, better positioning of the infant at the breast was the most frequent intervention. Challenges in a low dependency unit is shown in Fig. 2.

Inadequate production could very often be overcome by education and encouragement.

A unit with lactation counsellors demonstrated a higher rate of breastfeeding at discharge than one without, 90% (722/805) vs. 71% (421/590) *P* < 0.0001 chi-square.

Likewise, infants in the neonatal cubicles of the paediatric wards did not receive special attention and by the end of the project, were still to a large extent being fed dairy milk and formula.

### Human breast milk was donated

The amount of milk donated the first three years of the milk bank was on average 77 l per month. Each month almost 50 babies received DHM. It was experienced that donation was an incentive to establish milk production which later enabled more mothers to feed their infants mothers’ own milk. There were no economical transactions.

A milk kitchen and a rigid system were required for safe handling and optimal distribution of DHM. This was learned before the kitchens were in operation.

### Nursing care improved through knowledge and empowerment

Nurses acquired better observational and technical skills. Introducing “Nurses’ daily record” visualised their work and increased awareness of their impact. They reported themselves and were perceived as, more dedicated to and proud of their work, more attentive to and above all able and keen to act upon each infants’ and mothers’ special needs. Their empowerment made collaboration with other staff mutually more rewarding. KMC and breast or cup feeding by mothers (and later other caretakers) trained by the nurses eventually relieved time for improved standard care.

The measures for infection control were implemented. It was established acceptance of mutual obligation, irrespective of rank, to remind each other of hygiene.

### Referrals and bed strength increased

**Fig. 3:**
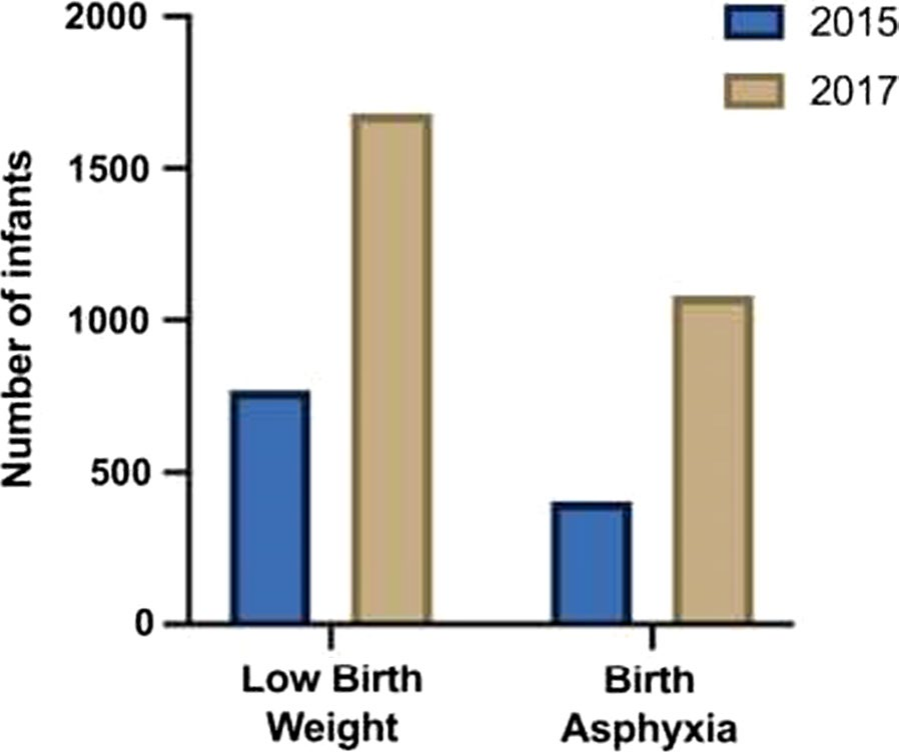
Example of diagnostic groups referred to JK Lone hospital. Low birth weight (LBW) and birth asphyxia (BA) increased from 770 and 406 in 2015, to 1680 and 1081 respectively in 2017. The most common diagnoses were jaundice and infection

During the period of the project, the number of infants referred to J K Lone increased continuously. This comprised also severe conditions (Fig. 3).

The neonatal unit gradually upgraded and expanded. In five years, the number of neonatal beds increased from 43 to 74 (and later to 150). There was still, however, inadequate capacity and neonates treated in the cubicles.

## Discussion

Optimal nutrition is a keystone in neonatal care. The multifaceted approach of this project, to make human milk production and distribution desirable and feasible increased feeding with human milk, hence improved newborn health.

### A comprehensive approach

The project was a collaboration between units with contrasting cultures and availability of resources. Hence, practiced neonatal care could not be replicated from one unit to the other. Endeavours were focused on customisation to location and time. This adaptable strategy to improve implementation has proved successful in similar projects [13]. Less hierarchal distribution of authoritative knowledge, as advocated by implementation strategists, was achieved by involvement of all levels and links in the decision chains and focus on mutual accommodation of knowledge [14]. Those implementing the practical interventions enjoyed intense support. These conditions were important assets of the project.

### Human resources

Having mothers close to their infants in the units was a major, difficult and crucial change. They provide the vital resource, milk, and are the final decision-makers in what and how to feed their infants. A mother’s physical contact with her infant increases hormone secretion enhancing milk production. Even if the infant is too weak, ill or premature to feed from the breast, tube or cup feeding with the preferred fresh milk requires the mother to be close by.

The number of infants not benefitting from at least trophic feeding is negligible [15].

Lactation counsellors were selected from postgraduates in non-science. To secure longevity and keep wages low, nurses were not preferred. They are often transferred between departments and are more prone to seek new challenges. As the skills of aiding breastfeeding improved among the nurses, more mothers could receive sufficient support from them, releasing time for counsellors to more challenging cases.

During the project, nurses were most empowered. Although the academic responsibility also regarding optimal and safe nutrition remained with the physicians, the driving force of neonatal care became more nurses centred, To improve the health of the infants, enabling them to receive enteral feeds and KMC (thus in addition to salubrious effects stimulate milk production) the interdisciplinary collaboration became even more important.

### DHM

This projects focus was guidance for mothers to provide enough milk for their infant. Human milk donation was a *means* not an end point. DHM as a gap filler, secures the infant good nutrition till mothers’ own milk is available. For the mothers the possibility of donating surplus milk encourages extraction to establish production for when their own infants will need it.

It soon became an honour to donate and the volume of DHM was surprisingly large. The Indians emphasised the importance of the milk being donated with positive feelings to do the recipient good. Accordingly, the counsellors always asked for donation of milk very gently and without any kind of pressure. As the recipients did not know the identity of the donors, the problem of milk kinship was rarely an issue [16].

In areas where mothers less often have access to a freezer to store their surplus expressed milk, the option of donation becomes relevant earlier. Milk produced early after birth is more energy dense and nourishing than milk produces later. In contrast to many high-income countries where the DHM mainly is from women who gave birth several weeks earlier, the Indian donors typically donated for a short while soon after delivery. Hence, this milk is probably even better suited for the newborns.

Our findings indicate that availability of DHM alone had less positive impact, than when combined with improved nursing care and lactation counselling. It is unlikely that the mothers unable to breastfeed their infants at discharge will manage this later. Initiating milk production is harder the longer it is delayed [17, 18].

The kind of problems preventing lactation and the increased lactation illustrates, in accordance with other studies how small efforts may have large impact [19].

### Hygiene

Upgrading hygiene was the first goal and throughout the project continuously subjected to high attention. Hand hygiene is the most effective way to prevent infections and thereby enhance the overall condition of the neonates [20]. Hence, it is plausible that this improvement was crucial for everything else.

### Sustainability

The department management had to be comfortable with the new arrangements in order to approve of the routines and stay responsible for required allocation of human and economic resources; everything from living facilities for mothers in the hospital to KMC-chairs, and most importantly, making it possible for nurses trained in the project to stay in the neonatal units, rather than being shifted. This secured a group capable of influencing the rest of the staff.

The most challenging parts of quality improvements often are initiation and sustainability [21]. We experienced that improving health literacy by providing a foundation through education for understanding the worth of measures made people wanting to make the effort, acquire the skills and change old practices [22]. Sustainability was strengthened in line with successful health promotion principles [23]; implementing hard to permute changes of infrastructure, educating and encouraging rather than deciding, involved decision making rather than premade solutions, and supporting training rather than merely demonstrating.

A plausible explanation for the increased number of referred patients is the hospitals’ better reputation regarding the infants` prognosis and the practice of involving parents. Some of these infants would otherwise go to private hospitals, others would not be treated. This complicates the comparison of data from different years. More importantly, cumulating and increasing the number of neonatal beds optimised care for more infants.

## Conclusion

The number of infants receiving optimal nutrition, human breast milk, increased during implementation of this quality improvement project.

Nutrition and quality of care of hospitalised neonates may be improved in a region with lower resource economy by focusing on human breast milk, education and optimization of human resources. Close collaboration at all hierarchical levels, development of customized methods and a broad approach are key factors for successful, viable development, possible through educational exchange of health care personnel.
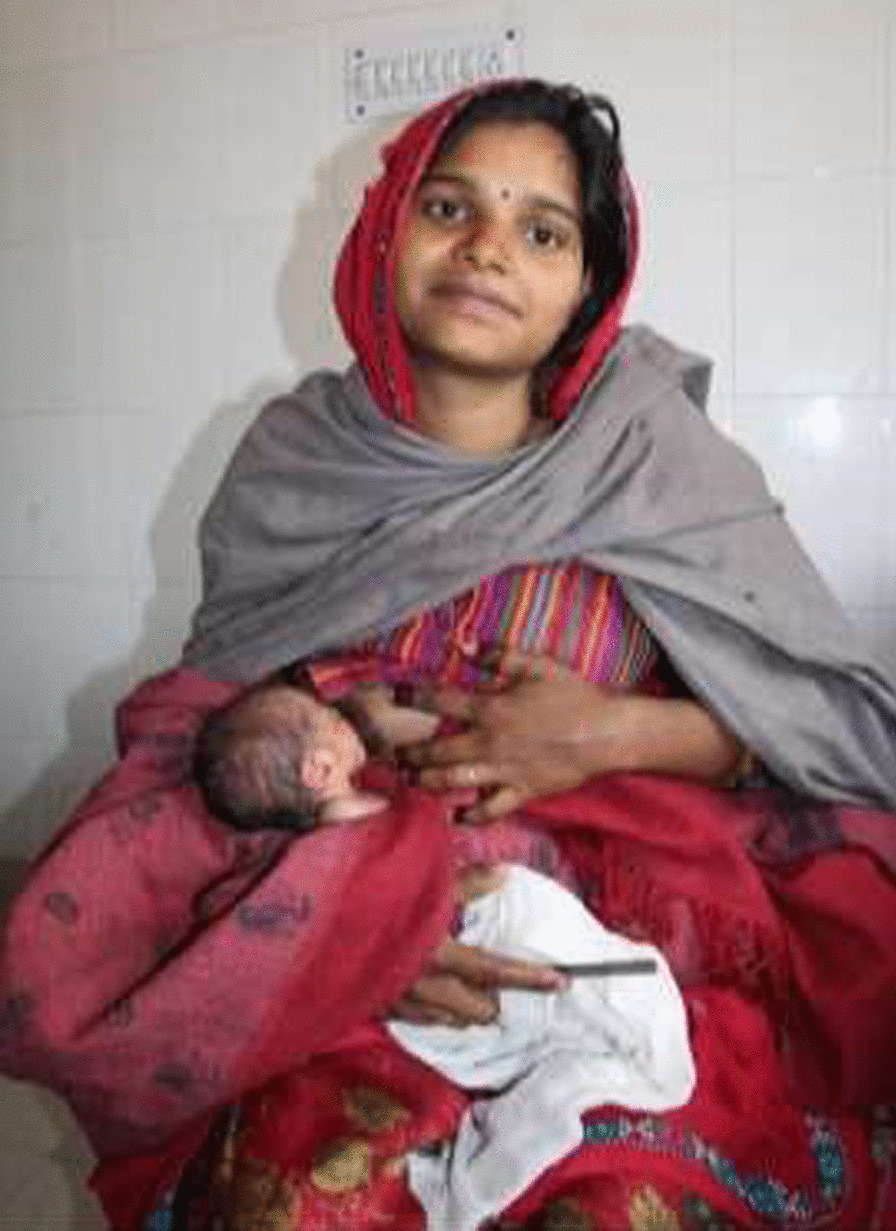


## Supplementary Information

**Additional file 1**. Participant report.

**Additional file 2**. Three days survey.

## Data Availability

All data and material are available from the author on reasonable request.

## Abbreviations

NMR: Neonatal mortality rate
DHM: Donated human breast milk
KMC: Kangaroo mother car
SNCU: Special newborn care unit
NICU: Neonatal intensive care unit
