# Correction to: Increased breastfeeding; an educational exchange program between India and Norway improving newborn health in a low- and middle-income hospital population

**DOI:** 10.1186/s41043-022-00306-w

**Published:** 2022-06-30

**Authors:** Kirsti Haaland, Sadasivam Sitaraman

**Affiliations:** 1 Department of Global Health, Oslo University Hospital, Ullevål, Kirkeveien 166, 0450 Oslo, Norway; 2 Department of Neonatal Intensive Care, Division of Paediatric and Adolescent Medicine, Oslo University Hospital, Ullevål, Kirkeveien 166, 0450 Oslo, Norway; 3 Sir Padampat. I.N.C.H. S.M.S. Medical College, Jaipur, India

## Correction to: Journal of Health, Population and Nutrition (2022) 41:16 https://doi.org/10.1186/s41043-022-00297-8

Following publication of the original article [1], the authors reported that the Acknowledgements section was missing. The Acknowledgements section is indicated hereafter.

**Acknowledgements**

The manuscript is based on the project “Oslo-Rajasthan Sick Newborn Care Training Programme” which was originally designed by The International Collaboration Unit, Oslo University Hospital and FK Norway, along with J K Lone Hospital, Sir Padam Pat Mother and Child Health Institute, Jaipur and Department of Medical Education and Department of Health and Family Welfare, Government of Rajasthan, India, with support from Royal Norwegian Embassy in New Delhi, India. Special thanks to the leadership of and to all health workers from SMS Medical College, India and Oslo University Hospital, Norway who made tremendous contributions to enable and develop the project. The author wishes to acknowledge sterling everyday contributions of the dedicated exchanged nurses and is particularly grateful to Dr Ramesh Choudhary for excellent collaboration and for his collection of data on exclusive breastfeeding after discharge.

The Acknowledgements section has been updated in this correction and the original article [1] has been corrected.
