# Supplementary material for: Increased breastfeeding; an educational exchange program between India and Norway improving newborn health in a low- and middle-income hospital population
Source: J Health Popul Nutr. 2022 May 3;41:16. doi: 10.1186/s41043-022-00297-8 (PMC9066889; doi:10.1186/s41043-022-00297-8)
Supplement: Supplementary file 1 — Additional file 1. Participant report. [file 41043_2022_297_MOESM1_ESM.docx]

# Oslo University Hospital

Ullevål Hospital

P O Box 4956 Nydalen

NO-0424 Oslo

Norway

Switchboard: +47 915 02770

# Participant report

1. **Main activities carried out in the period:**
2. **With reference to the aims and objectives of your work, how do you view your own contribution so far (give examples)?**
3. **What new procedures do you perform?**
4. **What new concepts have you learned?**
5. **What about your attitudes? Do you look differently on some areas of newborn care?**
6. **Have you experienced challenges during this period? If so, please specify. How can they be dealt with**?
7. **General work plan for the next period. What will be your priorities (work tasks) in the next period?**
8. **How are you integrated in the professional activities at your work-place? Do you have any suggestions for improvements?**
9. **How do you evaluate your social life in this period? Do you have any suggestions for improvements**?
10. **Other comments.**
