# Supplementary material for: Increased breastfeeding; an educational exchange program between India and Norway improving newborn health in a low- and middle-income hospital population
Source: J Health Popul Nutr. 2022 May 3;41:16. doi: 10.1186/s41043-022-00297-8 (PMC9066889; doi:10.1186/s41043-022-00297-8)
Supplement: Supplementary file 2 — Additional file 2. Three days survey. [file 41043_2022_297_MOESM2_ESM.docx]

**Three days survey form:**

| **Correct hand hygiene** |
| --- |
| Nurses |
| Doctors |
| **Not correct hand hygiene** |
| Nurses |
| Doctors |

| **Jewellery** |
| --- |
| Nurses |
| Doctors |
| **No jewellery** |
| Nurses |
| Doctors |

| **KMC** |
| --- |
| **Parent contact** |

| Breastfeeding |
| --- |
| **Breastmilk** |

| **Interrupted sleep** |
| --- |
| Nurses |
| Doctors |

| **Baby touch** |
| --- |
| Nurse |
| Junior Doctor |
| Senior Doctor |

| **Alarms not turned off** |
| --- |
| **Alarms turned off** |

| Response to cry |
| --- |
| No response to cry |

| Nested |
| --- |
| **No need for nesting** |
| **Not nested** |

| **Pain relief given** |
| --- |
| Nurses |
| Doctors |
| No pain relief given |

| Disinfection of skin |
| --- |
| Nurses |
| Doctors |
| No disinfection of skin |
| Nurses |
| Doctors |

| **Total temperature recording** |
| --- |

| **Total growth charts** |
| --- |

| **Total nurses daily record** |
| --- |
